# Comparing self-esteem and self-compassion: an analysis within the big five personality traits framework

**DOI:** 10.3389/fpsyg.2023.1302197

**Published:** 2023-12-14

**Authors:** Fan Yang, Chiaki Hagiwara, Tomomi Kotani, Joji Hirao, Atsushi Oshio

**Affiliations:** ^1^Graduate School of Letters, Arts and Sciences, Waseda University, Tokyo, Japan; ^2^Faculty of Letters, Arts and Sciences, Waseda University, Tokyo, Japan; ^3^Alphadrive Co., Ltd., Tokyo, Japan

**Keywords:** self-esteem, self-compassion, big five, correlations, partial correlations, cross-sectional

## Abstract

Self-esteem and self-compassion are two ways to relate to oneself. However, little is known about the similarities and differences between these two constructs. The current study used cross-sectional data from a Japanese sample to explore their relationship from a Big Five perspective. Results showed that differences between self-esteem and self-compassion appeared mainly in openness, agreeableness, and neuroticism. Specifically, self-esteem was uniquely associated with openness, and self-compassion was uniquely associated with agreeableness. Moreover, the negative correlation between self-compassion and neuroticism was larger than that between self-esteem and neuroticism. Implications and future directions are discussed.

## Introduction

Feeling well when we evaluate highly of ourselves, or just treating ourselves compassionately is enough? Previous studies investigate the different predictive roles of self-esteem and self-compassion in mental health, advocating that self-compassion could be one different way from self-esteem to relate to oneself (Neff and Vonk, 2009; Neff, 2023). By definition, self-esteem reflects an overall subjective evaluation of personal worth (Alessandri et al., 2015). On the other hand, self-compassion involves being open to and moved by one’s own suffering, experiencing feelings of caring and kindness toward oneself, taking an understanding, nonjudgmental attitude toward one’s inadequacies and failures, and recognizing that one’s own experience is part of the common human experience (Neff, 2003). However, how different (and similar) are these two constructs, and what do people with high self-esteem and self-compassion look like? Differences between these two constructs remain unclear. Big Five has been the dominant model within personality research, often represented as an effective taxonomy of psychological traits (Bainbridge et al., 2022). Attempts are made to adopt the Big Five personality traits as a conceptual framework to differentiate or organize various traits (Bainbridge et al., 2022). Therefore, the Big Five may also be a helpful framework that clarifies the relationship between self-esteem and self-compassion. Integrating self-compassion and self-esteem with the Big Five model can deepen our understanding of these constructs in relation to well-established personality dimensions. This integration allows for a more comprehensive analysis of how these traits interact with broader personality features. Understanding the relationship between self-compassion, self-esteem, and the Big Five traits can also inform the development of more tailored psychological interventions and therapies. For instance, interventions designed to enhance self-compassion or self-esteem can be adjusted to consider an individual’s specific personality profile. Self-compassion consists of three components: self-kindness, common humanity, and mindfulness (Neff, 2023). Self-kindness involves being emotionally available when life becomes difficult and responding to ourselves with warmth (Neff, 2023). Common humanity helps people to feel connected to others, remembering that everyone experiences suffering (Neff, 2023). Mindfulness is a type of balanced awareness that neither avoids nor exaggerates the discomfort of our present-moment experience (Shapiro et al., 2006; Neff, 2023), seeing experiences just as it is. Self-compassion, consisting of these components, is thus conceptually related to lower neuroticism, higher agreeableness, and higher openness. Likewise, higher self-esteem correlates with socially desired traits, such as higher agreeableness, higher openness, higher consciousness, higher extraversion, and lower neuroticism (Robins et al., 2001). Previous studies (Robins et al., 2001; Neff et al., 2007; Joshanloo and Afshari, 2011; Bainbridge et al., 2022) separately found that self-esteem and self-compassion negatively correlate with neuroticism and positively correlate with other domains of the Big Five. However, these results are not consistent and do not simultaneously put self-esteem and self-compassion into consideration. By simultaneously putting them into consideration, we can see the unique roles of self-esteem and self-compassion when ruling out their overlapping parts. In the current study, we expected self-esteem and self-compassion to correlate with openness, agreeableness, and neuroticism while exploring the different correlation patterns to the Big Five of self-esteem and self-compassion.

## Method

### Participants and procedures

Participants were recruited anonymously in the survey via Freeasy, a web research service provided by iBridge Co., Ltd., a Japanese company, in June 2023. The iBridge Co., Ltd. identifies potential participants for a study based on the study’s specific requirements, such as demographic criteria. In the present study, participants were required to live in Tokyo and above 18 years old. This identification process could involve filtering their participant database to find individuals who match the study’s criteria. Participants were recruited from a survey panel of approximately 4.5 million individuals. The participants familiarized themselves with the privacy policies of the study before participation. They were informed that they would be considered willing to participate if they chose to complete and submit the survey. Lastly, participants received non-monetary compensation from this online survey company upon successful completion and submission. All surveys that were fully completed were considered valid responses in the present study. Data on self-esteem, self-compassion, Big Five, age, and gender were collected by self-report measures from 504 Japanese participants (252 men and 252 women; *M*_age_ = 39.29, *SD* = 11.12). Correlational and partial correlational analyses were conducted to compare the Big Five features of self-esteem and self-compassion. All data analyses were conducted with IBM SPSS Statistics Version 29. We first conduct zero-order correlational analysis with self-esteem, self-compassion, and the Big Five. Then, partial correlational analyses conditioned on self-esteem or self-compassion would be conducted. Finally, we would include age and gender as controlled variables to carry out partial correlations.

### Measures

The reliabilities of measures used in the current study can be found in Table 1.

**Table 1:** Zero-order correlation coefficients, partial correlation coefficients, and descriptive statistics.

	Self-esteem		Self-compassion		*M*	*SD*	α	Inter-item *r*
	*r*	Partial *r*	*r*	Partial *r*				
Self-esteem			0.630^***^		25.046	5.624	0.854	
Self-compassion	0.630^***^				35.839	5.857	0.746	
Openness	0.376^***^	0.250^***^	0.304^***^	0.093^*^	7.579	2.073		0.244^***^
Conscientiousness	0.372^***^	0.193^***^	0.369^***^	0.187^***^	7.752	2.195		0.193^***^
Extraversion	0.401^***^	0.221^***^	0.385^***^	0.187^***^	7.645	2.476		0.294^***^
Agreeableness	0.239^***^	0.079	0.287^***^	0.180^***^	9.079	2.096		0.213^***^
Neuroticism	−0.455^***^	−0.121^**^	−0.602^***^	−0.457^***^	8.204	2.043		0.138^**^
Age	0.085	0.000	0.135^**^	0.106^*^	39.294	11.117		
Gender	0.038	0.093^*^	−0.055	−0.101^*^	1.500	0.500		

^*^p < 0.05, ^**^p < 0.01, ^***^p < 0.001. Numbers in the *r* column represent zero-order correlations between variables. In the partial *r* column under the self-esteem section, numbers are partial correlations between self-esteem and other variables when conditioned on self-compassion. In the partial *r* column under the self-compassion section, numbers are partial correlations between self-compassion and other variables when conditioned on self-esteem. In the inter-item *r* column, correlations between each two items of each big five domain. Regarding gender, men were coded as 1, and women were coded as 2.

#### Self-compassion

The 12-item Japanese version of the Self-Compassion Scale-Short Form (SCS-J-SF; Raes et al., 2011; Arimitsu et al., 2016). This measure employs a five-point Likert-type response format, ranging from 1 (almost never) to 5 (almost always). The SCS-SF measures self-compassion across three dimensions: self-kindness (e.g., “I try to be understanding and patient toward those aspects of my personality I do not like”), common humanity (e.g., “I try to see my failings as part of the human condition.”), and mindfulness (e.g., “When something painful happens, I try to take a balanced view of the situation”). A higher total score represents a higher self-compassion. The *α* of the SCS-J-SF in the current study reached 0.746, which is the same level as in the previous research (Arimitsu et al., 2016).

#### Self-esteem

The 10-item Japanese version of Rosenberg’s Self-Esteem Scale (Rosenberg, 1965; Sakurai, 2000) was adopted to measure self-esteem (e.g., “On the whole, I am satisfied with myself.”). This measure uses a four-point scale, from 1 (strongly disagree) to 4 (strongly agree). A higher total score indicates a level of higher self-esteem. The α of this scale in the current study was 0.854, which is the same level as in the previous study (Sakurai, 2000).

#### The Big Five personality traits

The Japanese version of the 10-Item Personality Inventory (TIPI-J; Gosling et al., 2003; Oshio et al., 2012) was used to measure the Big Five domains. That is openness (e.g., “I see myself as open to new experiences, complex”), conscientiousness (e.g., “I see myself as dependable, self-disciplined”), extraversion (e.g., “I see myself as extraverted, enthusiast”), agreeableness (e.g., “I see myself as sympathetic, warm”), and neuroticism (e.g., “I see myself as anxious, easily upset”). Each Big Five domain consists of two items, one of which is reverse-keyed. Participants were required to answer using a seven-point Likert-type scale, ranging from 1 = disagree strongly to 7 = agree strongly. A higher total score represents a higher level of each Big Five domain. The TIPI consists of five sub-scales, each composed of two items. Cronbach’s α coefficient is based on the average inter-item correlation of all items. When there are only two items, this average inter-item correlation is essentially the Pearson correlation coefficient between the two items. Therefore, a high correlation between these two items, instead of an α, indicates high internal consistency. On the other hand, TIPI attempts to measure the broad range implied by the subscales of the Big Five with just two items. From this perspective, a dilemma arises. That is, if the inter-item correlation is too high, the measurement range becomes limited. Correlations between each two items in the current study ranged from 0.138 to 0.294. This is consistent with suggestions from previous studies (Gosling et al., 2003; Oshio et al., 2012; Carciofo et al., 2016) that though the TIPI is less reliable compared with standard multi-item measures of the Big Five, it is recommended as a substitute, especially when research conditions dictate that a short measure be used.

## Results

Table 1 shows the zero-order and partial correlations and reliability of the Big Five, self-esteem, and self-compassion, as well as the age and gender of participants. There was a large size of significant positive correlation between self-esteem and self-compassion. Both self-esteem and self-compassion significantly and positively correlated with openness, conscientiousness, extraversion, and agreeableness while negatively correlated with neuroticism. More completed results of zero-order correlations can be found in Table S1.

When conditioning on self-compassion, self-esteem significantly and positively correlated with openness, conscientiousness, and extraversion and significantly and negatively correlated with neuroticism but did not significantly correlate with agreeableness. Regarding the partial correlations conditioned on self-esteem, self-compassion significantly and positively correlated with conscientiousness, extraversion, and agreeableness and significantly and negatively correlated with neuroticism. Though there was also a significant partial correlation between self-compassion and openness, it was quite weak. Supplementary Tables S2, S3 included more detailed results of partial correlations.

Further, we also calculated the partial correlations with conditioned on age, gender, and self-compassion (see Supplementary Tables S4, S5). Results showed that self-esteem significantly and positively partially correlated with openness, conscientiousness, and extraversion and significantly negatively with neuroticism but did not significantly correlate with agreeableness. When conditioning on age, gender, and self-esteem, self-compassion significantly and positively partially correlated with conscientiousness, extraversion, and agreeableness and significantly and negatively with neuroticism but did not significantly correlate with openness.

Putting together, differences between self-esteem and self-compassion appeared mainly in openness, agreeableness, and neuroticism. Specifically, self-esteem was uniquely associated with openness, and self-compassion was uniquely associated with agreeableness. Moreover, the negative correlation between self-compassion and neuroticism was larger than that between self-esteem and neuroticism.

## Discussion

Previous research advocated that self-compassion could be a substitute for self-esteem, but results regarding the comparison of self-compassion and self-esteem were not consistent and did not simultaneously put self-esteem and self-compassion into consideration. Therefore, the current study used a cross-sectional data set to compare the differences between self-compassion and self-esteem from a Big Five perspective. Our hypotheses that self-esteem and self-compassion correlate with openness, agreeableness, and neuroticism were supported. According to zero-order correlations, self-compassion was related to each Big Five domain in the same manner as self-esteem did. Furthermore, self-esteem and self-compassion correlated negatively with neuroticism and positively correlated with consciousness and extraversion, no matter in zero-order correlation or partial correlation. However, when controlling for age, gender, and self-compassion, there was no significant correlation between self-esteem and agreeableness. Likewise, there was no significant correlation between self-compassion and openness when controlling for age, gender, and self-esteem.

Our findings are consistent with previous studies and may provide theoretical and practical implications. First, self-compassion, but not self-esteem, was positively partially related to agreeableness, no matter whether age and gender were controlled or not. It is consistent with previous research that compared to self-esteem, self-compassion pays more attention to the need for relationships and community, leading to a higher correlation with agreeableness (Neff and Vonk, 2009; Neff, 2011). Second, self-esteem and self-compassion are both partially correlated with openness. However, the partial positive correlation between self-compassion and openness was quite small. Furthermore, when age and gender were controlled, only self-esteem partially correlated with openness. This is consistent with previous studies that high self-esteem individuals tended to ascribe socially desirable traits, like openness, to themselves, and this tendency partially mediated relations between the Big Five and self-esteem (Robins et al., 2001). The potential threat from social comparison to agreeableness may counterbalance the relationship between self-esteem and agreeableness (Neff, 2011). On the other hand, self-compassion includes less self-evaluation and social comparison, and the mindfulness component of self-compassion mainly focuses on internal feelings (Neff, 2023). Therefore, the correlation between self-compassion and openness may not be as large as between self-esteem and openness. Third, self-compassion puts more emphasis on emotionally comforting self when distressed. This is realized by noticing negative feelings without judging, thinking these experiences are shared by all human beings, and treating oneself with warm and supportive words and attitudes (Neff, 2023). These may explain why self-compassion correlated with neuroticism more than self-esteem did. Fourth, even when controlling for age and gender, both self-esteem and self-compassion still positively partially correlated with conscientiousness and extraversion to nearly the same degree. It would be interesting for future studies to examine whether self-esteem and self-compassion are interchangeable in interventions on conscientiousness and extraversion.

The cross-sectional Japanese sample used in the current study may limit our results. The current study aimed to explore the individual differences in the Big Five between self-esteem and self-compassion. However, cross-sectional data only provides between-person information and thus might lack the ability to detect within-person differences. Putting it in other words, a cross-sectional design only enabled us to capture what high self-esteem groups may be different from high self-compassion groups. We failed to see how the same person with high self-esteem or high self-compassion may be different. It is also an important issue because it is likely to provide more insight into the choice of self-esteem or self-compassion intervention for a specific person. Moreover, both self-esteem and self-compassion are regarded as culture-sensitive constructs (Schmitt and Allik, 2005; Neff, 2023). Therefore, it would be helpful if future research could adopt samples from different countries, using longitudinal or within-person experimental designs to replicate findings from the current study. Moreover, the current study was based on self-report methods. As aforementioned, self-esteem is likely to suffer from self-report biases (Robins et al., 2001). Future studies need to use different designs, for example, other-report self-esteem, to examine the results of the current study. Through different methods, the differences between state and trait self-compassion could also be explored (Chishima et al., 2022; Neff, 2023). In addition, while the TIPI-J is known for its brevity and ease of administration, it has been critiqued for its lower accuracy and depth compared to more comprehensive personality measures. Therefore, future studies may benefit from comparing self-esteem and self-compassion with a more complete version of the Big Five measure. Likewise, we only adopted a total score to index self-compassion, while self-compassion is thought to be a multi-dimensional construct. Future research may need to compare self-compassion with other constructs in a more detailed way.

Despite these limitations, the current study provided preliminary evidence that self-esteem and self-compassion are different from a Big Five personality perspective. By simultaneously considering self-esteem and self-compassion, the current study is likely to make a unique contribution to understanding how these two constructs relate to the Big Five personality traits. This helps reveal the distinct impact of self-esteem and self-compassion on interpersonal relationships and personal growth. Self-compassion may be more beneficial for agreeableness- or neuroticism-related interventions, while self-esteem may be more helpful for cultivating openness. Though self-esteem and self-compassion have several similar outcomes (Neff and Vonk, 2009), the current study suggested there may be different underlying mechanisms underlying these two constructs. It may be feasible to differentiate self-esteem and self-compassion by recognizing different personality features.

## Data availability statement

The raw data supporting the conclusions of this article will be made available by the authors, without undue reservation.

## Ethics statement

Ethical review and approval was not required for the study on human participants in accordance with the local legislation and institutional requirements. Written informed consent from the patients/ participants or patients/participants’ legal guardian/next of kin was not required to participate in this study in accordance with the national legislation and the institutional requirements.

## Author contributions

FY: Conceptualization, Data curation, Formal analysis, Methodology, Project administration, Validation, Visualization, Writing – original draft, Writing – review & editing. CH: Conceptualization, Methodology, Validation, Writing – review & editing. TK: Conceptualization, Investigation, Methodology, Resources, Writing – review & editing. JH: Conceptualization, Investigation, Methodology, Resources, Writing – review & editing. AO: Conceptualization, Methodology, Resources, Supervision, Writing – review & editing.
